# The United States Preventive Services Task Force (USPSTF) Guidelines vs. Electronic Health Record (EHR)-Based Screening: A Comparative Accuracy Study in Prostate Cancer

**DOI:** 10.7759/cureus.88863

**Published:** 2025-07-27

**Authors:** Kyung Hee Lee, Farrokh Alemi, Xia Wang, Mark Schwartz

**Affiliations:** 1 Recreation, Parks, and Leisure Science Administration, Central Michigan University, Mount Pleasant, USA; 2 Health Administration and Policy, George Mason University, Fairfax, USA; 3 Internal Medicine, Central Michigan University Medical Education Partners, Saginaw, USA; 4 Department of Population Health, NYU Grossman School of Medicine, New York City, USA

**Keywords:** artificial intelligence in medicine, ehr-based predictive model, electronic health records (ehr), preventative medicine, prostate cancer screening, united states preventive services task force (uspstf), uspstf guidelines

## Abstract

Background: The United States Preventive Services Task Force (USPSTF) provides age-based recommendations for prostate cancer screening. Such a single-criterion strategy can not only miss aggressive cancers that occur before the designated cut-off age, but also over-screen men whose cancers occur at a much older age. A multi-factorial model incorporating a wide spectrum of medical history may offer more accurate predictions. Such cancer prediction models may excel by incorporating diverse medical histories, including causal and non-causal conditions, as well as their chronological relationship with the onset of cancer.

Objective: This study aims to develop an AI (machine learning)-driven predictive model for prostate cancer based on patients’ medical history and compare its performance with traditional age-based criteria.

Methods: A predictive model was developed based on electronic health records (EHRs) from the *All of Us* database. A binary indicator for a prostate cancer diagnosis was established using SNOMED codes. Subsequently, a Least Absolute Shrinkage and Selection Operator (LASSO) logistic regression model was employed to examine the relationship between all prior health conditions and the cancer indicator, thereby identifying the most predictive features. Predictive performance was assessed using the area under the receiver operating characteristic curve (AUROC) and McFadden’s R².

Results: The EHR-based model achieved a 10-fold cross-validated McFadden’s R² of 0.36, significantly outperforming a model based on USPSTF eligibility criteria, which had an R² of 0.20. Validation using AUROC further demonstrated that the proposed model outperformed current screening criteria in terms of both sensitivity and specificity.

Conclusion: This study highlights the potential of personalized screening strategies and demonstrates that AI-driven prediction models based on EHR data can predict prostate cancer with better accuracy than existing age-based guidelines through non-invasive means. Such approaches may help reduce invasive diagnostic procedures due to unnecessary screening and improve early detection by focusing diagnostic efforts on those most at risk.

## Introduction

Prostate cancer remains a significant health concern in the United States, accounting for 29% of all cancer diagnoses in men [1]. The incidence of prostate cancer increased by 3% annually from 2014 through 2019, driven primarily by a rise in regional and distant-stage diagnoses, which have increased by approximately 4.5% per year since 2011 [2]. According to a 2024 report, approximately 299,010 new cases of prostate cancer and 35,250 related deaths were estimated for that year in the United States [3]. The incidence is highly age-dependent, with approximately 6 in 10 cases diagnosed in men aged 65 or older [4]. The rising incidence of late-stage diagnoses is particularly concerning, as it may hinder efforts to reduce mortality rates and underscores the critical importance of early detection and timely treatment.

The US Preventive Services Task Force (USPSTF) criteria for prostate cancer screening have evolved over time, reflecting a nuanced yet subjective approach to balance the benefits and harms of screening. Initially, in 2012, the USPSTF recommended against prostate-specific antigen (PSA)-based screening for prostate cancer in all men, citing concerns about overdiagnosis and overtreatment, which could lead to significant harms such as erectile dysfunction and urinary incontinence [5,6]. In 2017, the USPSTF revised its guidelines. In the current guidelines, which originated in May 2018, USPSTF endorses a subjective individual decision-making for men aged 55 to 69 years while still recommending against screening for men aged 70 and older due to the benefit-risk balance related to average life expectancy [7].

The current USPSTF prostate cancer screening eligibility requirement relies on age and thus does not consider other risk factors for cancer. Such a single-criterion strategy can not only miss aggressive cancer that occurred before the designated cut-off age, but also over-screen the men whose cancer occurred at a much older age. Several known risk factors for prostate cancer, such as chronic inflammation [8], alcohol consumption, vitamin D deficiency, and other lifestyle factors, are not considered by current age-based guidelines [5,9]. EHR-based predictive models can incorporate these established risk factors. EHR-wide predictor search can potentially identify novel risk factors. Further, clinical features preceding or co-existing with a cancer diagnosis may not pose a direct cause for the cancer, but an association can be detected between them due to a common predisposing factor.

## Materials and methods

Study design and data source

This study employed a retrospective, observational, case-control design, with cases defined as individuals diagnosed with prostate malignancy (identified via SNOMED code: 399068003) and controls as individuals without any malignancy diagnosis. Data from the All of Us Research Program [10], Controlled Tier V7, was utilized for this study. At the time of writing, the All of Us dataset includes 407,333 participants, of whom 287,012 have provided their EHRs. The inclusion criteria were adult males aged 18 and older who self-identified their gender as male. Among them, a total of 5,528 had a prostate cancer diagnosis. Prostate cancer stages could not be defined using SNOMED codes; therefore, cancer staging was not included in this study.

Sample size and variables

Sample size considerations indicated sufficient data availability for model development. A simulation-based power analysis, conducted in RStudio assuming a medium effect size of 0.25 and a significance level of <0.05, estimated a power of at least 80% across varying levels of temporal correlation among observations (low to medium). Using the approaches of Glueck and Muller [11,12], a covariance matrix was added to account for repeated measures within the final model.

The outcome variable of this study was primary malignant neoplasm of the prostate. A total of 25,683 SNOMED-CT codes, representing all diagnoses documented in inpatient and outpatient care environments, were used as binary independent predictors of cancer risks [13]. These independent variables include not only causal factors but also a broad range of potentially associated predictors to enhance accuracy. Unlike existing models, it incorporates rare diseases, chronic conditions, cancer precursors, viral infections, and prior cancer history factors that are often overlooked. While some of these predictors are non-causal, their inclusion improves risk stratification.

Analytical procedures

Statistical analyses were conducted interactively in R (v4.3.1) using a cloud-hosted Jupyter Notebook within the All of Us Researcher Workbench environment. We handled missing data using multiple imputation by chained equations (MICE) with the mice package in R [14]. To minimize circularity and ensure temporal validity in the predictive model, we excluded all data recorded after the initial diagnosis of prostate cancer. This decision was made to prevent the model from inadvertently learning from variables that are consequences of the diagnostic process or disease progression, which could artificially inflate performance metrics. To mitigate reverse causality, we included all disease codes recorded at least six months prior to the prostate cancer diagnosis as predictor variables. This six-month lead time minimizes confounding from the diagnostic process and increases the likelihood that selected variables reflect genuine early risk signals. Prostate cancer and its descendant codes were excluded from the set of independent variables. To control for confounding and data imbalance, we implemented one-to-one propensity score matching. This procedure created a balanced cohort by selecting a comparable control subject for each case based on age and race/ethnicity, effectively undersampling the non-cancer group. An age categorical variable (at the first diagnosis of cancer) was also included to compare current age-based USPSTF criteria for prostate cancer. Feature reduction was conducted in two stages. First, we applied Strong Rules, derived from the SAFE (Strong Rule-based Adaptive Feature Elimination) procedure [15], to eliminate features that were either unrelated to the outcome or exhibited high multicollinearity and instability across samples. These rules, based on univariate inner products between predictors and the outcome, efficiently screen out variables likely to have zero coefficients in the final model. While Strong Rules are not guaranteed to retain all active predictors, they have been shown to be highly effective in practice and rarely discard important features. To ensure correctness, we applied Karush-Kuhn-Tucker (KKT) condition checks during model fitting [16]. Second, we performed logistic LASSO (Least Absolute Shrinkage and Selection Operator) [17], which systematically shrinks coefficient estimates and sets those of weakly associated predictors to zero. The final set of predictors included variables with non-zero coefficients from the LASSO model, representing those with relatively strong and stable predictive value for prostate cancer risk under regularization.

Model validation

Model accuracy was validated using cross-validated McFadden’s R² and the area under the receiver operating characteristic curve (AUROC). A 10-fold cross-validation approach was employed, in which the data were randomly partitioned into ten equal-sized folds. The model was trained on nine folds and tested on the remaining fold, repeating this process ten times so that each fold served once as the test set. The average performance across folds was used to obtain stable and unbiased estimates of model accuracy.

## Results

The total study cohort comprised 407,333 patients, whose demographic and clinical characteristics are detailed in Table 1. Of this population, 360,918 (88.6%) were non-cancer patients, while 46,415 (11.4%) had a diagnosis of any cancer.

**Table 1:** Demographics of the Study Cohort by Cancer Status Percentages in parentheses refer to proportions of the total study population (N = 407,333). Female participants (N = 62) listed under prostate cancer were excluded from all inferential analyses, given the male-specific nature of the disease.

	Non-Cancer	Any Cancer	Prostate Cancer
Total	360,918 (88.6%)	46,415 (11.4%)	5,732 (1.4%)
Gender			
Female	219,981 (93.7%)	26,800 (11.8%)	62 (0.03%)
Male	133,329 (77.2%)	18,637 (10.8%)	5,528 (3.2%)
Age			
Age 18-44	129,648 (96.7%)	3,841 (2.9%)	13 (0.01%)
Age 45-64	130,028 (88.0%)	13,964 (9.4%)	821 (0.6%)
Age Over 65	93,634 (70.0%)	27,632 (20.6%)	4,756 (3.6%)
Race			
White Race	192,752 (86.4%)	32,141 (14.4%)	4,060 (1.8%)
Black Race	71,592 (91.6%)	6,002 (7.7%)	861 (1.1%)
Asian Race	13,338 (94.8%)	767 (5.4%)	57 (0.4%)
Ethnicity			
Hispanic	68,043 (92.2%)	5,508 (7.5%)	452 (0.6%)
Not Hispanic	278,788 (87.4%)	39,067 (12.3%)	5,105 (1.6%)

Our analysis focused on prostate cancer, which was identified in 5,732 individuals, accounting for 1.4% of the total cohort. To define the final cohort for analysis, we excluded cases with missing age information (N=142) and those identified as female (N=62), resulting in a final analytical cohort of 5,528 male patients over the age of 18.

The age distribution among all cases with available data (N=5,590) was as follows: 85.1% (N=4,756) were 65 years or older, 14.7% (N=821) were aged 45-64, and 0.2% (N=13) were aged 18-44. To address the data imbalance between the case group (cancer patients) and the control group (non-cancer patients), we applied one-to-one undersampling to create equal-sized groups. The sampling was based on cases of primary malignant neoplasm of the prostate.

Independent variables observed in fewer than 30 individuals were excluded based on near-zero variance criteria. [18], which are frequently implemented in standard machine learning preprocessing pipelines. Such features are known to contribute minimal variance and may distort model estimation or inflate noise when retained. After this filtering step, 9,405 variables remained. The SAFE procedure (correlation threshold = 0.5; variance threshold = 0.01) was subsequently applied, further reducing the predictor set to 807 by eliminating 8,598 variables. Finally, LASSO analysis identified 119 non-zero predictors for prostate cancer.

Table 2 provides a direct comparison of the structure and performance of two predictive models: the EHR-based model (Model 1) and the traditional USPSTF age-based criteria (Model 2). The table highlights a fundamental difference in their approaches. The USPSTF model relies on a single, categorical age bracket, whereas the EHR model employs a multi-factorial approach, incorporating a patient's age as a continuous variable alongside 119 statistically significant health conditions. This more detailed, personalized structure leads to a significant improvement in performance. McFadden’s R² quantifies the proportion of variance explained by a model, and was 0.363 for the EHR model, substantially higher than the 0.202 for the USPSTF model. This result demonstrates that the EHR-based model, leveraging a patient's detailed medical history, is a far more powerful and precise tool for predicting prostate cancer risk.

**Table 2:** Associated Factors and Age for Prostate Cancer *Inflammatory disorder of male genital organ (0.070); Benign prostatic hyperplasia without outflow obstruction (0.049); Benign neoplasm of colon (0.042); Prostatitis (0.037); Secondary erectile dysfunction (0.033); Gastroesophageal reflux disease (0.032); Strain of rotator cuff of shoulder (0.030); Enthesopathy of ankle AND/OR tarsus (0.029); Periinfarction syndrome (0.028); Pulmonary congestion and hypostasis (0.026); Inflammatory disorder (0.023); Melanocytic neoplasm (0.022); Tear of meniscus of knee (0.018); Complication of medical care (0.017); Hyperplasia of prostate (0.017); Conjunctivitis (0.017); Disorder of bursa of shoulder region (0.016); Coronary atherosclerosis (0.013); Degeneration of intervertebral disc (0.013); Disorder of lipid metabolism (0.013); Otitis media (0.012); Disorder of lung (0.011); Disorder of skin (0.011); Nodular prostate without urinary obstruction (0.009); Acute sinusitis (0.007); Hemorrhage of rectum and anus (0.006); Peripheral neuritis (0.005). The above factors are part of the identified risk factors.

Models	Model 1: EHR-Based Predictive (Associated Factors + Age)	Model 2: USPSTF Eligibility Requirement
Logistic Regression Coefficient	Age: 0.017, Risk Factors: See notes*	Age 55 to 69 years: 2.437
Intercept	-0.063	-1.457
Cross-Validated McFadden’s R²	0.363	0.202

**Figure 1:**
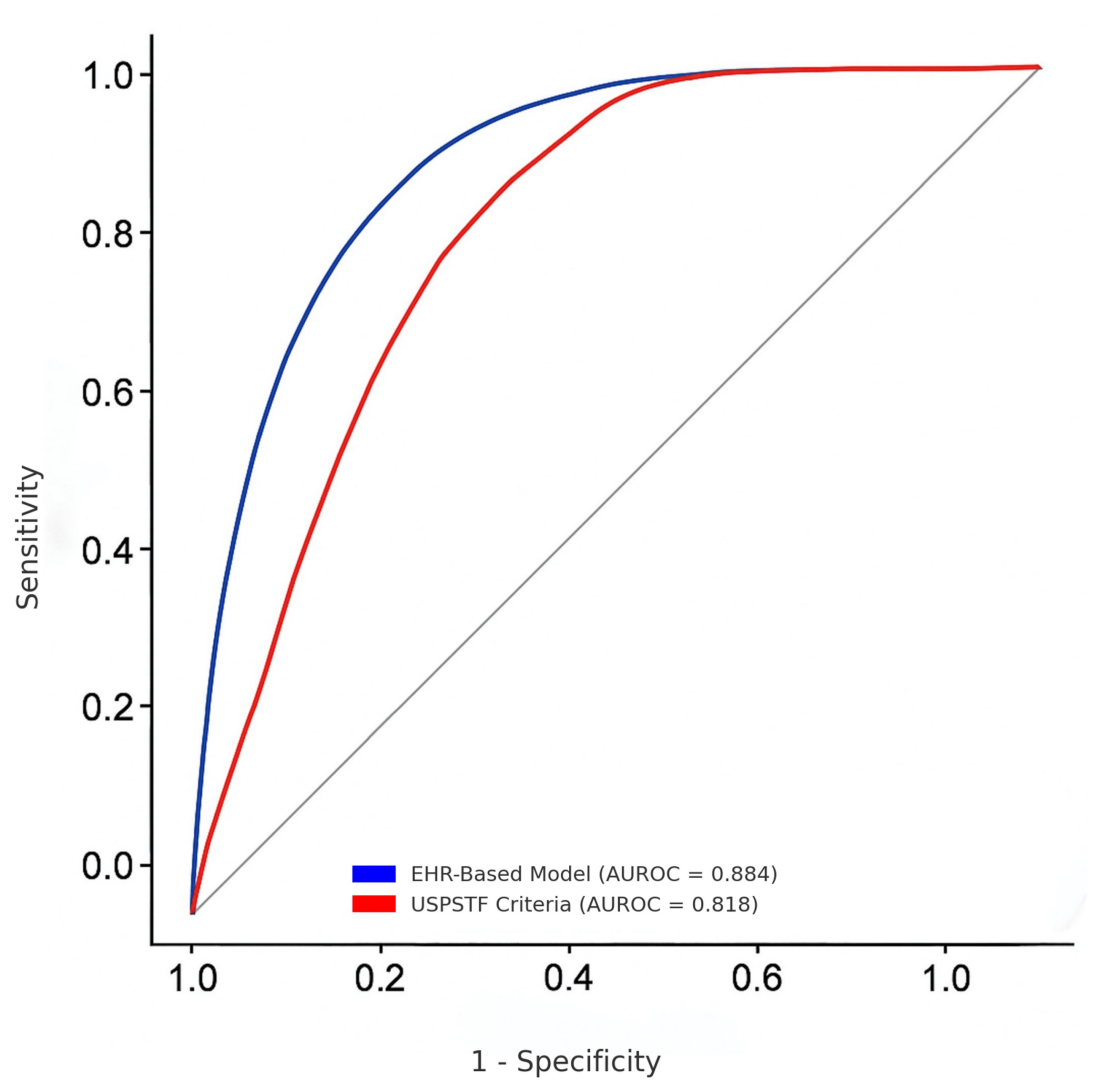
Comparison between USPSTF eligibility requirement and EHR-based predictive model USPSTF: United States Preventive Services Task Force; AUROC: area under the receiver-operating characteristic curve; EHR: electronic health record

The predictive accuracy of the two models is evaluated and compared using AUROC in Figure 1. The EHR-based model (blue curve) achieved an AUROC of 0.884, a value significantly higher than the 0.818 from the USPSTF criteria (red curve).

A higher AUROC value indicates a model’s enhanced ability to accurately distinguish between individuals with and without prostate cancer, and this result confirms the EHR-based model's substantially higher predictive accuracy. The receiver-operating characteristic (ROC) curve visually represents this performance difference by plotting the True Positive Rate (Sensitivity) on the y-axis against the False Positive Rate (1-Specificity) on the x-axis. The position of the EHR model’s curve above the USPSTF curve shows that it maintains a higher specificity (fewer false alarms) at any given level of sensitivity.

## Discussion

This study aims to develop an AI-driven predictive model for prostate cancer, specifically using machine learning, based on patients’ medical history, and to compare its performance with traditional USPSTF age-based screening criteria. It does not seek to explain the underlying etiology of prostate cancer. Rather, its primary goal is to enhance predictive accuracy by leveraging the breadth of real-world data available in EHRs. In the context of clinical screening, where early detection plays a decisive role in patient outcomes, predictive performance should take precedence over mechanistic interpretability. Even so, the model's ability to identify statistically significant predictors that are consistent with prior medical literature reinforces the validity of its data-driven design. Among the top-ranked variables were several well-established risk factors: (1) inflammatory disorders of male genital organs; (2) benign prostatic hyperplasia without outflow obstruction; (3) benign neoplasm of the colon; (4) prostatitis; and (5) secondary erectile dysfunction [19-26]. The successful replication of these associations strengthens confidence in the model’s construct validity.

Our proposed model deliberately includes both causal and non-causal predictors to maximize overall predictive performance [27,28]. While some variables may not have a direct biological relationship with prostate cancer, they may function as proxies for shared pathways or heightened diagnostic activity. For example, erectile dysfunction may prompt more frequent prostate evaluations, thereby increasing opportunities for cancer detection. These indirect signals, whether causal or not, improve the model's ability to stratify risk accurately, an important consideration in screening frameworks that prioritize sensitivity and specificity.

Although concerns remain that predictive models might contribute to overdiagnosis of indolent or nonlethal prostate cancers, refined prediction models may help address this issue by improving both sensitivity and specificity. In particular, multidimensional EHR-based models can potentially detect clinically relevant cases among individuals who fall outside of traditional screening criteria. In our study population, we observed 821 prostate cancer cases in men aged 45 to 64, and 13 in those under 45. Both groups are currently ineligible under the USPSTF screening guidelines. This distribution underscores the need to reevaluate rigid age-based thresholds and consider more flexible, risk-based approaches. Notably, prostate cancer diagnosed at younger ages is more likely to be aggressive, emphasizing the importance of timely detection in this subgroup [29,30]. At the same time, a more precise model may reduce overtreatment by limiting unnecessary interventions in low-risk individuals.

Despite the inclusion of a large number of predictors, the model is designed with practical implementation in mind. It can be embedded into existing EHR systems as a passive risk alert or clinical decision support feature. Moreover, AI-powered, patient-facing applications may extend the reach of such tools to settings beyond the clinic, empowering individuals to engage with their own risk data. As with laboratory diagnostics, the complexity of input variables need not impede usability; predictive models should be viewed as analytical extensions of clinical reasoning, supporting personalized care through data-driven insights.

Limitations and future directions

There has always been a concern that over-diagnosing indolent low-grade prostate cancer can lead to increasing morbidity without a positive impact on mortality. Prostatitis and prostate hyperplasia may have intensified scrutiny for the prostate and led to more diagnoses of low-grade prostate cancer. Due to the absence of detailed cancer stage information in the All of Us dataset, the study is unable to facilitate more action and/or accurate diagnosis toward higher-grade prostate cancer, for which timely intervention is crucial to reduce mortality. A future in-depth study incorporating cancer stages will achieve this goal. Another limitation is the lack of detailed analysis regarding differences in incidence rates and risk factors among diverse racial and ethnic groups [31]. While the study sample includes a variety of races and ethnicities, a comprehensive analysis of incidence rates and risk factors across these specific groups was beyond the scope of the current study. Future studies should delve deeper into these demographic distinctions to develop more personalized screening strategies tailored to specific populations. External validation of this model in other cohorts is warranted.

## Conclusions

This study demonstrates that a predictive model leveraging high-dimensional data from comprehensive EHRs significantly outperforms conventional age-based guidelines in identifying individuals at risk for prostate cancer. By integrating a broad range of established and novel predictors, our model offers a more accurate and personalized approach to screening eligibility, improving both sensitivity and specificity compared to current criteria. This machine learning-driven approach holds considerable promise for optimizing early detection efforts, potentially reducing the need for unnecessary invasive procedures while also offering economic advantages by lowering related costs and time. It enhances diagnostic focus on those who need it most. Ultimately, our findings advocate for a paradigm shift toward more precise, AI-driven prostate cancer screening strategies to improve patient outcomes.

## References

[1] Abodunrin F, Adeoye O, Masih D, Nelson N, Silberstein PT, Tupper C (2023). Late-stage prostate cancer and associated socioeconomic and demographic factors: A National Cancer Database Study. Am Soc Clin Oncol.

[2] Siegel RL, Miller KD, Wagle NS, Jemal A (2023). Cancer statistics, 2023. CA Cancer J Clin.

[3] (2025). American Cancer Society. Cancer Facts & Figures 2024. https://www.cancer.org/research/cancer-facts-statistics/all-cancer-facts-figures/2024-cancer-facts-figures.html.

[4] Washington C, Goldstein DA, Moore A, Gardner U Jr, Deville C Jr (2022). Health disparities in prostate cancer and approaches to advance equitable care. Am Soc Clin Oncol Educ Book.

[5] US Preventive Services Task Force (2018). Screening for prostate cancer: US Preventive Services Task Force Recommendation Statement. JAMA.

[6] Presti J Jr, Alexeeff S, Horton B, Prausnitz S, Avins AL (2020). Changes in prostate cancer presentation following the 2012 USPSTF screening statement: Observational study in a multispecialty group practice. J Gen Intern Med.

[7] Leapman MS, Wang R, Park H (2022). Changes in prostate-specific antigen testing relative to the revised US Preventive Services Task Force recommendation on prostate cancer screening. JAMA Oncol.

[8] Cai L, DeBerardinis RJ, Zhan X, Xiao G, Xie Y (2024). Navigating electronic health record accuracy by examination of sex incongruent conditions. J Am Med Inform Assoc.

[9] Zhang B, Shi H, Wang H (2023). Machine learning and AI in cancer prognosis, prediction, and treatment selection: A critical approach. J Multidiscip Healthc.

[10] (2025). National Institutes of Health. All of Us Research Program. https://allofus.nih.gov/.

[11] Glueck DH, Muller KE (2003). Adjusting power for a baseline covariate in linear models. Stat Med.

[12] Muller KE, Lavange LM, Ramey SL, Ramey CT (1992). Power calculations for general linear multivariate models including repeated measures applications. J Am Stat Assoc.

[13] (2025). All of Us Research Hub. Data Browser [Internet]. https://databrowser.researchallofus.org/.

[14] Deng Y, Chang C, Ido MS, Long Q (2016). Multiple imputation for general missing data patterns in the presence of high-dimensional data. Sci Rep.

[15] Tibshirani R, Bien J, Friedman J, Hastie T, Simon N, Taylor J, Tibshirani RJ (2012). Strong rules for discarding predictors in lasso-type problems. J R Stat Soc Series B Stat Methodol.

[16] Tibshirani RJ (2013). The lasso problem and uniqueness. Elect J Stat.

[17] Gallieri M (2016). Principles of LASSO MPC. Lasso-MPC - Predictive Control with ℓ1-Regularised Least Squares.

[18] Kuhn M, Johnson K (2013). Applied predictive modeling. https://link.springer.com/book/10.1007/978-1-4614-6849-3.

[19] Huang L, LaBonte MJ, Craig SG, Finn SP, Allott EH (2022). Inflammation and prostate cancer: A multidisciplinary approach to identifying opportunities for treatment and prevention. Cancers.

[20] Chakravarty D, Ratnani P, Huang L (2022). Association between incidental pelvic inflammation and aggressive prostate cancer. Cancers.

[21] Dai X, Fang X, Ma Y, Xianyu J (2016). Benign prostatic hyperplasia and the risk of prostate cancer and bladder cancer: A meta-analysis of observational studies. Medicine (Baltimore).

[22] Sunkavalli M, Yen R, Kothari N, Sitrin M, Pomakov O (2010). Increased risk of colorectal adenomas in patients with prostate cancer. Official J Am Coll Gastroenterol.

[23] Roberts RO, Bergstralh EJ, Bass SE, Lieber MM, Jacobsen SJ (2004). Prostatitis as a risk factor for prostate cancer. Epidemiology.

[24] Doat S, Marous M, Rebillard X (2018). Prostatitis, other genitourinary infections and prostate cancer risk: Influence of non-steroidal anti-inflammatory drugs? Results from the EPICAP study. Int J Cancer.

[25] Lin WY, Chang YH, Lin CL, Kao CH, Wu HC (2017). Erectile dysfunction and the risk of prostate cancer. Oncotarget.

[26] Ramspek CL, Steyerberg EW, Riley RD, Rosendaal FR, Dekkers OM, Dekker FW, van Diepen M (2021). Prediction or causality? A scoping review of their conflation within current observational research. Eur J Epidemiol.

[27] Shmueli G (2025). To explain, to predict, or to describe: Figuring out the study goal [Commentary on "On the uses and abuses of regression models" by Carlin and Moreno-Betancur]. Stat Med.

[28] Dilixiati D, Kadier K, Laihaiti D, Lu JD, Azhati B, Rexiati M (2023). The association between sexual dysfunction and prostate cancer: A systematic review and meta-analysis. J Sex Med.

[29] Sartor O (2020). Why is prostate cancer incidence rising in young men?. Cancer.

[30] Chatterjee A, Fan X, Slear J (2024). Quantitative multi-parametric MRI of the prostate reveals racial differences. Cancers (Basel).

[31] Salinas CA, Tsodikov A, Ishak-Howard M, Cooney KA (2014). Prostate cancer in young men: an important clinical entity. Nat Rev Urol.

